# TrkB Receptor Antagonism Enhances Insulin Secretion and Increases Pancreatic Islet Size in Rats Fed a Cafeteria-Style Diet

**DOI:** 10.3390/biomedicines13010126

**Published:** 2025-01-08

**Authors:** Jorge Agustín Velasco-Gutierrez, Elena Roces de Alvarez-Buylla, Sergio Montero, Alejandrina Rodríguez-Hernández, Saraí Limón Miranda, Karmina Martínez-Santillan, María del Rosario Álvarez-Valadez, Mónica Lemus, Alejandra Flores-Silva, Adolfo Virgen-Ortiz

**Affiliations:** 1 Centro Universitario de Investigaciones Biomédicas, Universidad de Colima, Colima 28045, Colima, Mexico; 2 Facultad de Medicina, Universidad de Colima, Colima 28040, Colima, Mexico; 3 Facultad Interdisciplinaria de Ciencias Biológicas y de Salud, Departamento de Ciencias Químico Biológicas y Agropecuarias, Unidad Regional Sur, Universidad de Sonora, Navojoa 85800, Sonora, Mexico

**Keywords:** cafeteria-style diet, BNDF-trkB pathway, neurotrophins, pancreas, insulin, metabolic syndrome, obesity, ANA-12 drug, HOMA index

## Abstract

**Background:** In recent years, the role of neurotrophins and their receptors in peripheral tissues has been of great interest. At a metabolic level, the brain-derived neurotrophic factor (BDNF) and its receptor trkB have been reported to participate in insulin secretion from the pancreas in response to increases in circulating blood glucose. **Objetive:** To determines the role of the BDNF-trkB pathway in insulin secretion and pancreatic morphology in rats fed a cafeteria-style diet for 16 weeks. **Methods:** For the study, male rats of the Wistar strain were divided into three groups as follows: (1) control group (standard diet), (2) CAF group (cafeteria-style diet) and (3) CAF group treated with ANA-12 (TrkB receptor antagonist). After 4 months of intervention, the glucose and insulin tolerance curves, serum insulin levels, body fat and hematoxylin-eosin staining pancreas were evaluated. **Results:** The results showed that the cafeteria-style diet induced an increase in the amount of body fat, alterations in the glucose tolerance curve, increased insulin circulation levels, increased HOMA indices and increased pancreatic islet size. The antagonism of the trkB receptor in the rats fed a cafeteria-style diet enhanced some effects such as the accumulation of body fat and insulin secretion and induced a greater increase in the pancreas islet size. **Conclusions:** Under conditions of cafeteria-style diet-induced obesity, the antagonism of the BDNF-trkB pathway had no enhanced effect on the increase in insulin secretion or pancreatic islet size.

## 1. Introduction

The neurotrophins family includes modulators of synaptic plasticity [1,2] and consists of four proteins as follows: nerve growth factor (NGF), brain-derived neurotrophin factor (BDNF), neurotrophin 3 (NT-3) and neurotrophin 4/5 (NT-4/5). The growth factors exert their effects by binding to the family of receptor tyrosine kinases (trk receptor A, B and C) and binding to the p75 neurotrophin receptor (p75NTR) [3]. The function of the neurotrophins family and its receptors are widely studied in brain-modulating neuronal survival, development, function and plasticity [4]. The presence of trk A, B and C receptors as well as p75NTR in non-neuronal peripheral tissues such as the pancreas has resulted in great interest in their functional implication in these tissues and in the development of metabolic diseases where there is a large information gap. Studies over the past decade using BDNF knockout mice have shown that they can develop obesity through a deficit in satiety [5]. The development of hepatic steatosis has also been observed in this model [6]. Another interesting study in muscle-specific BDNF knockout mice showed that ingesting a high-fat diet exacerbated the development of obesity, insulin resistance, intramyocellular lipid deposition and mitochondrial dysfunction [7]. The selective deletion of BDNF in the hypothalamus of adult mice resulted in increased food intake and obesity [8,9]. In addition, this BDNF-TrkB signaling is known to be an important pathway involved in β-cell survival through the activation of the IRS1/2, PI3K and Akt pathways, which promote the expression of genes encoding proteins responsible for cell survival. The downstream signaling cascade of BDNF is similar to that of insulin-like growth factor-1 (IGF-1), including p-CAMK and MAPK. This pathway is involved in the expression of pro-survival genes; thus, BDNF prevents β-cell exhaustion [10]. The activation of the BDNF/TrkB/CREB pathway reduces hepatic gluconeogenesis, glucose levels, leptin and food intake, inducing hepatic insulin signal transduction, elevating the number of glycolytic fibers in skeletal muscle and protecting against pancreatic β-cell loss in diabetes mellitus [11]. It has also been reported that there is an association between reduced BDNF levels and impaired glucose metabolism in patients with type 2 diabetes mellitus [10,12].

The present study focuses on studying the relationship between neurotrophins and their receptors in the regulation of circulating blood glucose, in particular BDNF and its receptor trkB. Experiments in obese mice fed a high-fat diet demonstrated that BDNF administration improves glucose tolerance and reduces non-fasting insulin levels [13,14]. These findings were among the first to suggest an involvement of BDNF in blood glucose regulation at the systemic level. A recent study using immunoblot demonstrated the presence of the trkB receptor in mouse pancreas. In the same work, mice were developed with trkB receptor deletion in which neither beta cell mass nor insulin content was affected; however, at the functional level it caused a negative effect on glucose tolerance, which did not affect insulin tolerance but decreased glucose-induced insulin secretion. In pancreatic islets of wild-type mice, glucose stimulation increases insulin secretion, and in the case of the trkB receptor, it deleted islet glucose stimulation and increased insulin secretion. Interestingly, BDNF potentiated insulin secretion in wild-type mouse isolates (a similar effect was observed in human islets) and in trkB receptor-deleted isolates, it did not potentiate insulin secretion [15]. These experiments support the idea that BDNF and its receptor trkB are important in insulin secretion from pancreatic islets pre-stimulated with glucose in healthy mice; in addition, there is evidence that BDNF signaling promotes beta cell growth and insulin secretion [16]. However, little is known about the involvement of the pancreatic trkB receptor in systemic glucose regulation in other non-physiological conditions. In this context, the aim of our study was to evaluate the involvement of the trkB receptor in pancreatic islet morphology, circulating insulin levels and systemic glucose regulation among rats fed a cafeteria-style diet, a model widely used to study metabolic disorders [17,18,19]. The cafeteria-style diet involves rodents eating the same unhealthy but tasty ultra-processed products consumed by humans (e.g., bacon, muffins and cookies), which are readily available in supermarkets. The CAF diet model, due to its palatability, texture and odor promotes overconsumption by mimicking the behavior of humans with regard to orosensory properties [20]. The CAF diet is defined as hypercaloric and hyperlipidic, and it is mainly composed of fats and sugars [17].

ANA-12 [21], a high-selectivity antagonist to the trkB receptor was used in this study as an experimental strategy to evaluate the role of the trkB receptor in pancreas and glucose regulation. Several studies have demonstrated the usefulness of ANA-12 in blocking BDNF-trkB signaling to assess the role of this pathway in different tissues and cellular and functional processes [22,23,24]. Finally, there have been no reports of toxicity associated with this trkB receptor antagonist.

## 2. Materials and Methods

### 2.1. Animal Care

Male Wistar rats (weight 299 ± 4 g, approximately 8 weeks of age) were used, maintained under standard bioterium conditions (12/12 h light/dark cycles, relative humidity maintained between 45 and 60%, a room temperature of 24 °C ± 1 °C, with free access to water and food). All animals were treated according to the international ethical principles for the use of experimental animals. This research project and the use of rats for this study was approved by the Internal Committee for the Care and Use of Laboratory Animals of the University Center for Biomedical Research, University of Colima (Date: 27 January 2022, approval code: 2022-1CD).

### 2.2. Experimental Design

Thirty rats were randomly assigned to three experimental groups as follows: (1) control group with standard diet (CTRL, *n* = 10), (2) cafeteria-style diet group (DCAF, *n* = 10) and (3) cafeteria-style diet plus ANA-12 treatment group (DCAF-ANA12, *n* = 10).

The diet intervention protocol was for a period of 16 weeks (the period necessary for the development of metabolic alterations, insulin resistance and cardiovascular alterations as reported in previous studies [25]). During this time, the control group only consumed the standard diet (rodent diet 5001: 29% kcal proteins, 13% kcal fat, 58% kcal carbohydrates, 5.2% kcal fiber) and water, with free access to both. The cafeteria-style group, for the same time, received a regionalized menu of ultra-processed products with a high sugar and fat content (see Table 1) and one product per day (for example, during the first week, the foods provided were day 1 = chocolate-filled sweet bread; day 2 = turkey breast ham; day 3 = chocolate-filled wafer cookies; day 4 = cheesy chips; day 5 = white chocolate and strawberry jam-filled pastry; day 6 = leg ham; day 7 = jelly beans). The design of the cafeteria-style diet was based on a previous study [18]. The products for this diet were purchased in supermarket stores located in the city of Colima, Mexico. The third experimental group received a cafeteria-style diet for 10 weeks, and in the following 6 weeks, the group received a cafeteria-style diet and a daily dose of ANA-12 (0.5 mg/kg body weight) intraperitoneally [21]. ANA-12 was purchased at MedChemExpress (Monmouth Junction, NJ, USA).

### 2.3. Determination of Glucose and Insulin Tolerance Curves

After 16 weeks of intervention, the glucose tolerance curve was derived for all experimental groups under fasting conditions by administering a glucose load of 1 g/kg body weight intraperitoneally and monitoring the glucose concentration in peripheral blood samples at different times (0, 15, 30, 60, 90 and 120 min). For the insulin tolerance curve, an insulin load of 0.75 IU/kg body weight was administered and glucose concentrations in peripheral blood were recorded over a period of two hours. The peripheral blood sample was taken from the tail tip of the rat after asepsis, and the measurement of glucose concentration was performed with Accu-Chek Instant^®^ portable equipment (Roche products, Mexico City, Mexico). The results of the tolerance curves were expressed as the area under the curve (AUC).

### 2.4. Sample Collection

Under anesthesia with sodium pentobarbital at a dose of 45 mg/kg body weight, administered intraperitoneally, the pancreas was dissected, extracted and placed in 10% buffered formalin fixative solution (Sigma-Aldrich Corporation, Saint Louis, MO, USA) for subsequent analysis. Then, 7–10 mL of blood was collected by cardiac puncture for further analysis. Finally, the rats were sacrificed by exsanguination.

### 2.5. Determination of Insulin Concentration by the ELISA Method

The blood samples obtained from all experimental groups were allowed to coagulate and the serum was then separated and used for insulin determination. To quantify the insulin concentration, the rat insulin ELISA kit (Invitrogen, Waltham, MA, USA) was used, and the protocol recommended by the manufacturer was followed. This method for quantifying the amount of insulin present in the samples generates a colorimetric reaction whose absorbance is read in a microplate spectrophotometer (Thermo Electro Corporation Multiscan Ascent, Thermo Fisher Scientific, Waltham, MA, USA) at 450 nm. The calculation of the insulin concentration in the samples was performed from a standard curve of reference concentrations with an R^2^ = 0.99. The results were expressed in µIU/mL.

### 2.6. Histological Analysis

Pancreatic tissue samples previously fixed in 10% buffered formalin were embedded in paraffin to make 5 μm-thick sections, which were subjected to staining using the hematoxylin-eosin technique. Images of the stained slides were then taken (Camera Axiocam Icc1 coupled to microscope Axio LabA1, Carl Zeiss, Gottingen, Germany) and a morphological analysis was performed to determine the size of the islets with AxioVision Imagining software (version 4.8; Carl Zeiss, Gottingen, Germany).

### 2.7. Statistical Analysis

All data were expressed as mean ± standard deviation. Normality analysis was performed using to Shapiro–Wilk test. The three experimental groups were compared with a one-factor analysis of variance (ANOVA) followed by multiple pairwise comparisons with Tukey’s test for post hoc analysis. Statistical significance was considered for values of *p* < 0.05. The graphs and analyses were performed with GraphPad Prism version 8.0.1 software.

## 3. Results

### 3.1. Analysis of Body Weight and Total Fat

**Figure 1:**
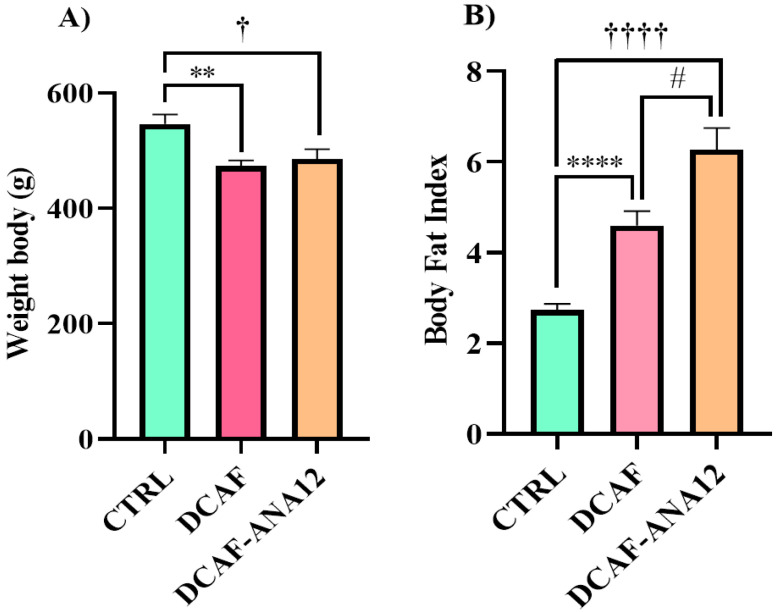
Effect of chronic consumption of cafeteria-style diet on (**A**) body weight and (**B**) body fat index in male rats. TrkB receptor antagonism enhanced the increase in body fat. CTRL, control group with standard diet; DCAF, cafeteria-style diet group; DCAF-ANA12, cafeteria-style diet group and ANA-12 drug treatment. The groups were compared with one-way ANOVA and Tukey’s test for post hoc analysis. Differences between groups were significant at *p* < 0.05. Symbols asterisk (CTRL vs. DCAF, ** *p* < 0.01; **** *p* < 0.0001), dagger (CTRL vs. DCAF-ANA12, † *p* < 0.05; †††† *p* < 0.0001), hashtag (DCAF vs. DCAF-ANA12, # *p* < 0.05).

At the end of the 16 weeks of the diet and treatment intervention, body weight variations were observed in the three experimental groups, but the cafeteria-style groups showed a lower body weight, which was statistically significant (CTRL, 546 ± 17 g; DCAF, 473 ± 10 g; DCAF-ANA12, 485 ± 18 g). After sacrificing the rats, the abdominal, renal and gonadal fat were removed and weighed. The amount of total fat was normalized with respect to body weight and expressed as the body fat index. The body fat index increased significantly in the DCAF group compared to the CTRL group (CTRL group, 2.73 ± 0.14; DCAF group, 4.60 ± 0.32). The ANA-12 treatment group (6.27 ± 0.48) had a significantly higher body fat index than the DCAF group and the CTRL group. Chronic cafeteria-style diet feeding induces a loss of body weight but an increase in the amount of body fat. Treatment with ANA-12 enhanced the accumulation of body fat (Figure 1).

### 3.2. Glucose and Insulin Tolerance Curves

**Figure 2:**
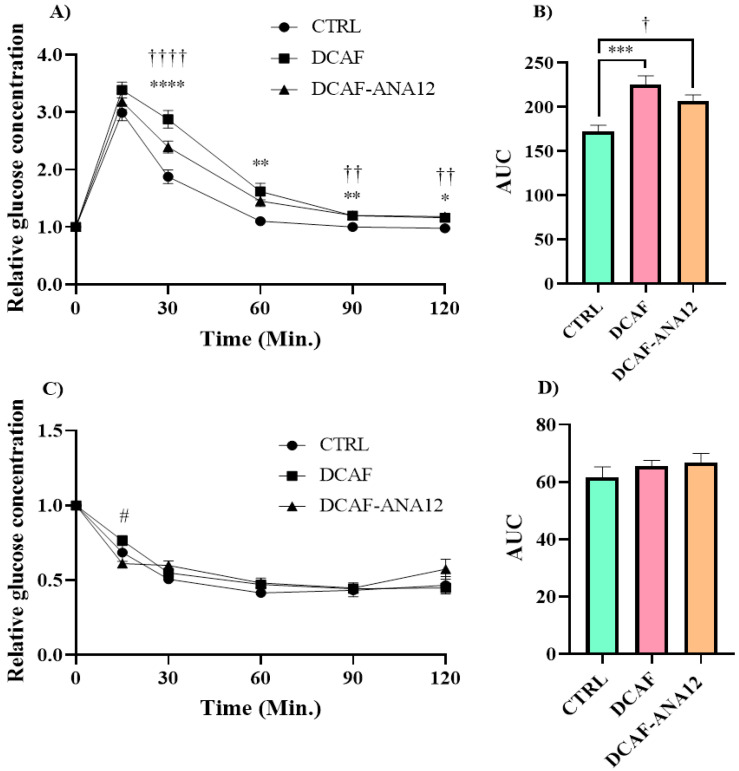
Glucose (**A**,**B**) and insulin tolerance (**C**,**D**) curves in rats fed cafeteria-style diet and treated with the trkB receptor antagonist. AUC, area under the curve; CTRL, control group with standard diet; DCAF, cafeteria-style diet group; DCAF-ANA12, cafeteria-style diet group and ANA-12 drug treatment. The groups were compared with one-way ANOVA and Tukey’s test for post hoc analysis. Differences between groups were significant to *p* < 0.05. Symbols asterisk (CTRL vs. DCAF, * *p* < 0.05; ** *p* < 0.01; *** *p* < 0.001; **** *p* < 0.0001), dagger (CTRL vs. DCAF-ANA12, † *p* < 0.05; †† *p* < 0.01; †††† *p* < 0.0001), hashtag (DCAF vs. DCAF-ANA12, # *p* < 0.05).

Fasting blood glucose values were not significantly modified by the cafeteria-style diet (CTRL group, 100 ± 2 mg/dL; DCAF group, 99 ± 2 mg/dL; DCAF-ANA12 group, 96 ± 1 mg/dL). The analysis of the tolerance curves showed that the cafeteria-style diet induced a dysregulation in glucose tolerance, and the AUC of the DCAF group was significantly higher than that of the CTRL group (225 ± 10 vs. 172 ± 7). The same effect was observed in the DCAF-ANA12 group with respect to the CTRL group (AUC, 206 ± 7 vs. 172 ± 7) (Figure 2).

There were no significant differences between the DCAF and DCAF-ANA12 groups in glucose (A,B) and insulin tolerance (C,D) curves. The cafeteria-style diet negatively altered glucose tolerance and trkB receptor antagonism with ANA-12 administration, and it did not show a significant effect on glucose disturbance. On the other hand, no significant differences were observed in the insulin tolerance curves of the three groups studied (AUC, CTRL 62 ± 4; DCAF 66 ± 2; DCAF-ANA12 67 ± 3) (Figure 2).

### 3.3. Insulin Secretion Analysis and Pancreatic Histology

**Figure 3:**
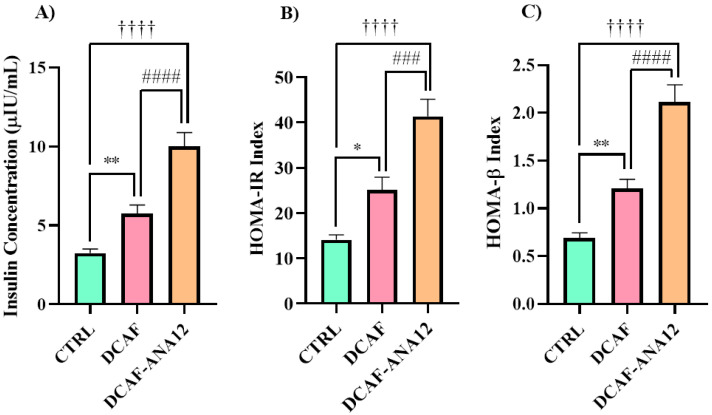
Serum insulin levels (**A**) and HOMA indices (**B**,**C**) of rats fed a cafeteria-style diet and treated with ANA-12 to antagonize the trkB receptor. CTRL, control group with standard diet; DCAF, cafeteria-style diet group; DCAF-ANA12, cafeteria-style diet group and ANA-12 drug treatment. The groups were compared with one-way ANOVA and Tukey’s test for post hoc analysis. Differences between groups were significant to *p* < 0.05. Symbols asterisk (CTRL vs. DCAF, * *p* < 0.05; ** *p* < 0.01), dagger (CTRL vs. DCAF-ANA12, †††† *p* < 0.0001), hashtag (DCAF vs. DCAF-ANA12, ### *p* < 0.001; #### *p* < 0.0001).

The cafeteria-style diet induced a significant increase in insulin secretion of 92% with respect to the rats fed a standard diet; meanwhile, for the group of rats that were fed a cafeteria-style diet with the addition of ANA-12 administration, insulin secretion increased 234% with respect to the control group. When comparing the two groups that were fed a cafeteria-style diet, for the group treated with the ANA-12 drug, insulin secretion showed a significant increase of 74% (see Figure 3). The chronic ingestion of a cafeteria-style diet generates an increase in fasting insulin secretion. The antagonism of the trkB receptor with ANA-12 enhanced insulin secretion in rats fed a cafeteria-style diet.

HOMA-IR and HOMA-β indices increased with the chronic ingestion of a cafeteria-style diet, and when rats received treatment with the ANA-12 antagonist, these indices were even higher (Figure 3). These values show that the cafeteria-style diet promotes the development of insulin resistance and pancreatic hyperfunction by increasing insulin secretion, and trkB receptor antagonism with ANA-12 exacerbated the effects of the cafeteria-style diet.

**Figure 4:**
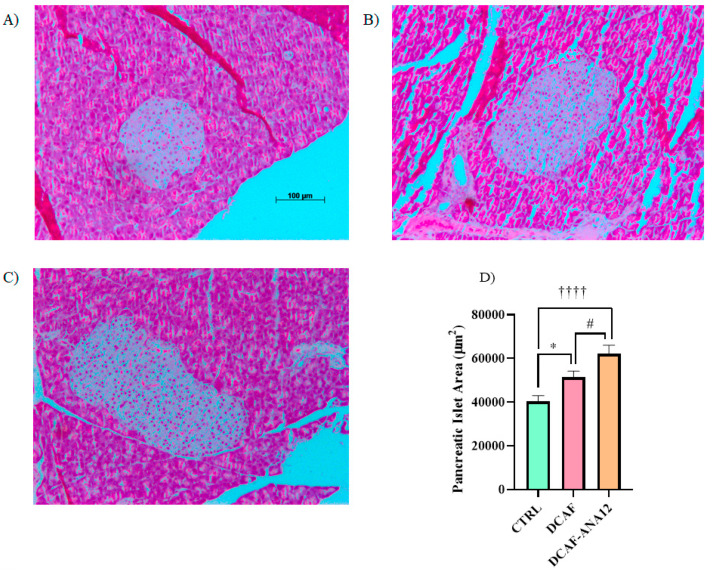
Illustrative images of pancreatic islets stained with hematoxylin-eosin. (**A**) CTRL, control group with standard diet; (**B**) DCAF, cafeteria-style diet group; (**C**) DCAF-ANA12, cafeteria-style diet group and ANA-12 drug treatment; (**D**) Comparative analysis of islet size. The groups were compared with one-way ANOVA and Tukey’s test for post hoc analysis. Differences between groups were significant to *p* < 0.05. Symbols asterisk (CTRL vs. DCAF, * *p* < 0.05), dagger (CTRL vs. DCAF-ANA12, †††† *p* < 0.0001), hashtag (DCAF vs. DCAF-ANA12, # *p* < 0.05). Images (**B**,**C**) have the same scale as shown in (**A**).

Pancreas sections were stained with hematoxylin-eosin to analyze their islet size. The measurement of pancreatic islets, shown in Figure 4, revealed that chronic supplementation with a cafeteria-style diet induced an increase in islet size compared to islets from rats supplemented with the standard diet. Furthermore, in rats fed a cafeteria-style diet, when the ANA-12 antagonist was administered, the increase in islet size was potentiated. The quantitative analysis of pancreatic islet area showed that the chronic feeding of a cafeteria-style diet significantly induced an increase in islet area (28%) compared to the control group, and a greater increase was observed in rats treated with ANA-12, following a cafeteria-style diet (71%) (Figure 4).

## 4. Discussion

The main objective of this research was to evaluate the involvement of the trkB receptor in the regulation of circulating blood glucose and pancreatic islet size in rats fed a cafeteria-style diet for 16 weeks. The study involved the comparison of the following three experimental groups: (1) group with standard diet, (2) group with cafeteria-style diet intake and (3) group with cafeteria-style diet intake plus administration of ANA-12, a specific trkB receptor antagonist. The main findings of this study were that the cafeteria-style diet caused an increase in body fat, disturbances in glucose tolerance, increased insulin secretion and pancreatic islet area as well as insulin resistance. Moreover, the trkB receptor antagonism with ANA-12 in rats fed a cafeteria-style diet enhanced body fat accumulation, circulating insulin levels and insulin resistance, and it increased pancreatic islet area. These results suggest that the trkB receptor is important in the control of body fat accumulation and the regulation of insulin secretion when a cafeteria-style diet is consumed for prolonged periods. These data are consistent with previous studies using BDNF knockout murine models that reported the development of obesity and increased insulin resistance [5,7].

In both humans and rodents, the pancreas plays a crucial role in the regulation of blood glucose through insulin secretion. In both species, the cells responsible for this function are beta cells, which uptake circulating glucose through GLUT-1 transporters in humans and GLUT-2 in rodents, and then metabolize it and produce ATP. The increase in the ATP/ADP ratio blocks the outflow of potassium through ATP-sensitive potassium channels, depolarizes the membrane activating voltage-dependent calcium channels which allow calcium to enter the cell and this calcium promotes the exocytosis of insulin-loaded vesicles favoring secretion into the bloodstream [26]. Insulin secretion is activated by a post-prandial increase in glucose concentrations, a classical mechanism referred to as glucose-stimulated insulin secretion (GSIS). This also suggests that free fatty acids potentiate GSIS through their binding FFAs to the highly expressed FFAR1 receptors on beta cells, and this downstream activation increases intracellular calcium levels and the activation of protein kinase C (PKC) and protein kinase D (PKD1), which increase insulin exocytosis [27,28,29]. The cafeteria-style diet is high in lipids and sugars, and the constant intake of these foods with free access activates the GSIS mechanism and mechanisms that enhance insulin secretion, which explains the effects of the cafeteria-style diet observed in this work.

The increase in insulin secretion and HOMA index values induced by the consumption of a cafeteria-style diet for long periods of time agrees with previous studies, which have also reported increased insulin gene expression in the pancreas [30]. The cafeteria-style diet has been reported to induce body fat accumulation [31,32,33,34] and skeletal muscle atrophy [35]. Insulin resistance and body fat accumulation promote a compensatory increase in beta cell mass and pancreatic islet size as observed in this study and others [36].

In addition, the BDNF-trkB signaling pathway is widely studied in the brain, where it participates in the regulation of satiety and body weight control at the level of the hypothalamus [8,9]. However, recent studies demonstrated the presence of the trkB receptor in beta cells and showed evidence of the direct action of BDNF on the trkB receptor that elicited a glucose-stimulated increase in insulin secretion. In vitro and in mice experiments with trkB receptor deletion show the importance of BDNF-trkB pathway activation in regulating insulin secretion and cell size in beta cells [7,10]. Interestingly, a study carried out in bottlenose dolphins characterized by hyperinsulinemia found, by immunolocalization, the presence of NGF and BDNF in the pancreas, which may be involved in the hyperinsulinemia that is observed in dolphins [37].

In our study, the blockade of the trkB receptor in rats with hyperinsulinemia induced by a cafeteria-style diet did not reduce insulin levels, but rather further increased hyperinsulinemia and pancreatic islet size, suggesting that another pathway is activated that is also involved in insulin secretion in the pancreas.

In this context, it has been reported that NGF and its receptor trkA are expressed in the pancreas [38]; in addition, in response to increased glucose, NGF stimulates insulin secretion in beta cells through trkA [39]. It is also known from previous studies that in obese states, NGF levels increase, and by binding to the receptor trkA could increase insulin secretion and generate hyperinsulinemia in obesity conditions [40].

In our study, the cafeteria-style diet increased body fat content, and if we also consider that in a state of obesity it has been reported that BDNF levels decrease [41], it is possible that by antagonizing the trkB receptor with ANA-12, hyperinsulinemia was enhanced by the activity of NGF binding to trkA receptor. Recently, it was also shown that NGF participates in beta-cell maturation [42], which could also explain the increase in islet size observed in our study.

In conclusion, the intake of 16 weeks of a cafeteria-style diet induced the following effects:

(1) Increased body fat.
(2) Increased insulin resistance.
(3) Increased circulating insulin levels and pancreatic islet size.
(4) TrkB receptor antagonism enhanced the above effects.
(5) The effects observed by antagonizing the trkB receptor suggest that the BDNF-trkB pathway is not the only mechanism involved in insulin secretion in obese conditions.

**Figure 5:**
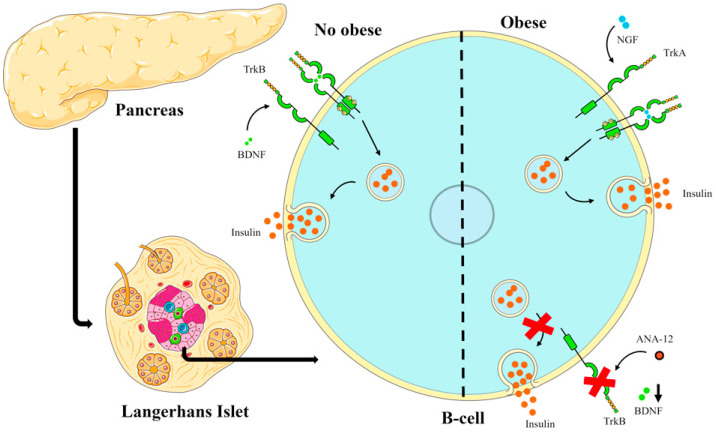
Scheme illustrating the action of BDNF in non-obese and obese conditions on insulin secretion. In obesity-inducing diets such as the cafeteria-style diet, the BDNF-trkB pathway may be suppressed and the NGF-trkA pathway may play a key role in hyperinsulinemia in obese conditions.

With the data obtained in this study and the results of other studies [37,38,39,40], the following scheme was elaborated to explain the possible role that BDNF and NGF may play in pancreatic islets, as shown in Figure 5.

### Limitations

- Some studies show gender differences in the physiological and metabolic responses and the oxidative stress induced by a cafeteria-style diet in rodents [19,43,44], therefore, it would be important to evaluate the effects of BDNF-trkB pathway blockade on glucose and pancreas regulation in females in future studies.
- Evaluating NGF levels and trkA receptor expression in the pancreas of rats fed a cafeteria-style diet with and without trkB receptor antagonism in future studies would improve our understanding of the involvement of both neurotrophins in the control of insulin secretion by pancreatic beta cells.

From a broader perspective, this study opens a line of research for designing future experiments focused on better understanding the participation of different neurotrophins and their receptors in physiological and non-physiological conditions such as obesity.

## Figures and Tables

**Table 1:** Composition of products used in the cafeteria-style diet.

	Macronutrients (% kcal)			Total % kcal
Products	Carbohydrates	Proteins	Fat	
Chocolate bars	44.74	4.39	51.32	100.45
Turkey sausages	9.16	27.49	63.35	100.00
Gummies	92.33	7.55	0.00	99.88
Chips	49.73	6.21	44.15	100.09
Sweet wholemeal bread	52.13	7.62	40.26	100.00
Sausages for roasting	22.50	20.00	56.25	98.75
Cookies with sweet filling	61.78	4.56	34.03	100.36
Sweet bread with vanilla	44.63	4.92	50.43	99.98
Chocolate-filled cupcakes	60.10	5.18	34.74	100.03
Sausages	17.78	20.00	60.00	97.78
Vanilla marshmallows	95.12	4.88	0.00	100.00
Sweet bread with raisins	68.31	8.90	22.72	99.93
Pineapple marmalade-filled pastries	66.93	4.85	28.14	99.92
Turkey ham	36.00	56.00	9.00	101.00
Strawberry and chocolate marshmallows	80.44	2.07	17.41	99.92
Salted chips	39.41	3.99	56.56	99.96
White chocolate and strawberry jam-filled pastries	57.97	3.65	38.38	100.00
Leg ham	15.15	62.14	22.72	100.00
Jelly beans	95.16	4.84	0.00	100.00
Vanilla cookies	66.93	4.85	28.14	99.92
Sweet bread filled with chocolate	59.59	4.74	35.72	100.05
Turkey breast ham	27.56	40.34	31.76	99.66
Wafer-type cookies with chocolate filling	52.94	3.17	43.93	100.04
Cheesy chips	40.91	4.04	55.03	99.98
Chocolate cookies filled with white chocolate	59.53	3.51	37.00	100.04
Turkey Sausages	11.23	27.37	61.58	100.18
Strawberries and vanilla marshmallows	95.12	4.88	0.00	100.00
Chocolate chip cookies	57.09	4.53	38.46	100.09

## Data Availability

Data are contained within the article.
